# Role of Titanium Dioxide-Immobilized PES Beads in a Combined Water Treatment System of Tubular Alumina Microfiltration and PES Beads

**DOI:** 10.3390/membranes13090757

**Published:** 2023-08-25

**Authors:** Sungtaek Hong, Sungwoo Park, Jin Yong Park

**Affiliations:** Department of Environmental Sciences & Biotechnology, Hallym University, Chunchon 24252, Republic of Korea; hst1642@naver.com (S.H.); sw12park@naver.com (S.P.)

**Keywords:** titanium dioxide, photocatalysis, water air backwashing, ceramic, microfiltration, water treatment

## Abstract

The membrane process has a limit to the decay of various pollutants in water. To improve the problem, the roles of backwashing media and titanium dioxide (TiO_2_) photocatalyst-immobilized-polyethersulfone (PES) beads’ concentration were investigated in a combined system of tubular alumina MF and the PES beads for advanced drinking water treatment. The space between the outside of the MF membrane and the module inside was filled with the PES beads. UV at a wavelength of 352 nm was irradiated from outside of the acryl module. A quantity of humic acid and kaolin was dissolved in distilled water for synthetic water. Water or air intermittent backwashing was performed outside to inside. The membrane fouling resistance after 3 h process (R_f,180_) was minimum at 30 g/L of the PES beads for water backwashing, and at 40 g/L for air backwashing when increasing the PES beads from 0 to 50 g/L. The irreversible membrane fouling resistance after physical cleaning (R_if_) was at the bottom at 5 g/L of the PES beads for water backwashing, which was 3.43 times higher than minimal at 40 g/L of the PES beads for air backwashing. The treatment effectiveness of turbidity increased when increasing the PES beads’ concentration from 0 to 50 g/L; however, it reached a maximum at 98.1% at 40 g/L and 99.2% at 50 g/L for water and air backwashing, respectively. The treatment effectiveness of UV_254_ absorbance, which was dissolved organic matter (DOM), increased dramatically when increasing the PES beads; however, it reached a peak of 83.0% at 40 g/L and 86.0% at 50 g/L for water and air backwashing, respectively. Finally, the best PES beads’ concentration was 20~30 g/L to minimize the membrane fouling; however, it was 50 g/L to remove pollutants effectively. The water backwashing was better than the air at treating DOM; however, the air backwashing was more effective than the water at removing turbid matter and reducing membrane fouling.

## 1. Introduction

In a membrane water treatment system, membrane fouling generally occurs by the adsorption–precipitation of inorganic and organic composites on the membrane inside and surface and leads to a decline in the treated water flux, to a rise in membrane cleaning prices, and to a decrease in membrane life. Approaches for dropping membrane fouling stay insufficient, which is the chief interruption in the prosperous application of a membrane separation system; however, significant improvement has been made in membrane fouling regulation [1,2]. A principal component of membrane fouling is natural organic material (NOM) in low-pressure membrane water treatment. Many defending processes to constrain NOM fouling have been achieved and broadly certified, such as mineral oxide adsorption, carbon adsorption, ion exchange, oxidation, and coagulation [3]. A novel adsorbent, such as heated aluminum oxide powder, was hired in a completely automated pilot water treatment process to eliminate NOM in the surface water [4]. 

Among a number of photocatalysts, TiO_2_, which was utilized in this research, is superior, because it is non-toxic, photochemically stable, highly active, and inexpensive [5,6]. TiO_2_ has been applied to various membrane water treatment research. Cu^2+^/ZnO/TiO_2_ nanocomposites were prepared by the sol-gel method and applied to decay benzene, toluene, and xylene in oilfield-produced water [7]. Immobilized TiO_2_ mesocrystals were manufactured on sanded glass and tested by the photo-degradation of various dyes [8]. A packed-bed photocatalytic reactor with immobilized TiO_2_ on glass beads was tried to eliminate seven pharmaceuticals [9].

Backwashing/backpulsing occasionally can be installed to get rid of reversible fouling for membrane fouling control [10,11]. Chemical cleaning is required to eliminate irreversible fouling and to return the membrane performance, in which case that the membrane performance reduces by 50–60% [12]. To prevent the membrane fouling in this work, intermittent air or water backwashing was applied in the membrane water treatment system.

Nowadays, the combined system of membrane separation and photo-oxidation by UV radiation can successfully solve the membrane fouling limitation [13,14]. The combined system not only preserves the profits of each technology but also produces synergistic effects to overwhelm the restrictions of such a technique. Moreover, pollutants such as NOM can be oxidized by UV radiation, and organic substances are lessened somewhat by regulating the staying time in the reactor. Otherwise, the restriction of membrane separation is just a selective barrier where only molecules lesser than their pore size can be separated. In summary, the combined system is able to raise the photo-oxidation effectiveness and obtain excellent effluent quality. Furthermore, the impact of UV radiation on the nano-hybrid PES-NanoZnO membrane by removal effectiveness and flux has been debated [15]. Moreover, an estimation of the treatment effectiveness of surface water was researched in a combined water treatment system of various advanced oxidation processes and ultrafiltration (UF) [16]. In this work, a combined water treatment system of a ceramic membrane and TiO_2_-immobilized PES beads with UV radiation was utilized for excellent water quality.

Ceramic membranes applied in this study usually have a triple upper price than polymeric membranes containing comparable membrane surface area. Nevertheless, those have numerous advantages, which include a long lifetime, chemical, thermal, and mechanical resistance. The ceramic membranes were cost-effective, paralleled with polymeric membranes, owing to greater permeating flux, and essentially a long-lasting lifetime [17]. Presently, reformed and enhanced ceramic membranes have been utilized comprehensively in water or wastewater treatment globally [18,19]. At the laboratory scale, the effect of the appearance of soluble algal organic matter (AOM) on the membrane fouling was inspected for a 7-channel tubular ceramic MF membrane [20]. The influence of the collaboration between aquatic humic materials and the AOM coming from microcystis aeruginosa was studied on the membrane fouling of a ceramic MF [21]. A flat-sheet ceramic membrane was applied to municipal wastewater treatment by a high-rate membrane bioreactor for competent recovery of organic substances [22].

Photo-oxidation has various benefits which are a comprehensive kind of utilization, small energy consumption, and great effectiveness. Especially, for non-biodegradable organic contaminants, the mineralization of organic composites to small inorganic molecules by oxidization of most of them can be explained by the mechanism of the photo-oxidation process. It is also one of the outstanding technologies of advanced water treatment systems. For these inspirations, the photo-oxidation method, which was applied in this study, has been utilized broadly [23,24,25,26,27]. Moreover, the degradation of humic acid (HA), which was included in an artificial solution utilized in this study via photoelectrocatalysis (PEC) process, and resultant disinfection byproduct formation potential (DBPFP) were inspected, and the PEC process was revealed to be active in the decreasing of dissolved organic carbon concentration [28].

The influence of water backwashing in the combined water treatment system of TiO_2_ coated-PP beads and multi-channel ceramic MF or tubular carbon fiber UF with UV radiation were researched by our group [29,30]. Also, portions of adsorption and photo-oxidation in a combined water treatment system of tubular carbon fiber UF and pure PP beads with UV radiation and water backwashing was studied by our team [31]. 

In this research, the roles of backwashing media (water, air) and titanium dioxide (TiO_2_) photocatalyst-immobilized PES beads’ concentration were investigated in a combined system of tubular ceramic MF and PES beads for advanced drinking water treatment. Replacing DOM and turbid substances, a persistent quantity of humic acid (HA) and kaolin was dissolved in distilled water. This research was the unique application of TiO_2_-immobilized PES beads [32,33], which were manufactured by a dissimilar method to prepared TiO_2_-coated-PP beads [29,30], and UV radiation to inspect the effect of backwashing media and PES beads’ concentration in the combined water treatment system. A combined module was constituted of the TiO_2_-immobilized PES beads and the ceramic MF. The PES beads were packed between the space of the acryl module case and the ceramic MF.

## 2. Materials and Methods

The tubular ceramic (NCMT-7231, pore size 0.1 μm) MF membrane, manufactured by Nanopore Inc. (Seoul, Republic of Korea), was utilized in this work. The characteristics of the tubular ceramic MF are summarized in Table 1. TiO_2_ photocatalyst-immobilized PES beads of 1.2~1.4 mm were employed in this research, and were prepared with the non-solvent induced phase inversion method for the immobilization of the catalyst TiO_2_ in E. Drioli’s research team [32,33]; their detailed information is summarized in Table 2. 

Replacing soluble natural organic substances and very small inorganic particles in natural water sources such as a river or lake, a persistent quantity of HA and kaolin was dissolved in water prepared by distillation equipment. In this study, it was applied as artificial feed water. Two UV lamps (F8T5BLB, Sankyo, Japan) irradiated UV with 352 nm, which was the most effective wavelength to decompose DOM from the acryl module outside and to reduce DOM by photo-oxidation. 

To remove the turbid matters and DOM, the TiO_2_-immobilized PES beads were packed in the gap between the ceramic MF outside and the module inside. Additionally, for protection of the PES beads loss out of the module, a 100 mesh (0.150 mm) sieve, which was much less than the 4–6 mm size of the PES beads used in this study, was attached to the outlet of the combined module. 

**Figure 1:**
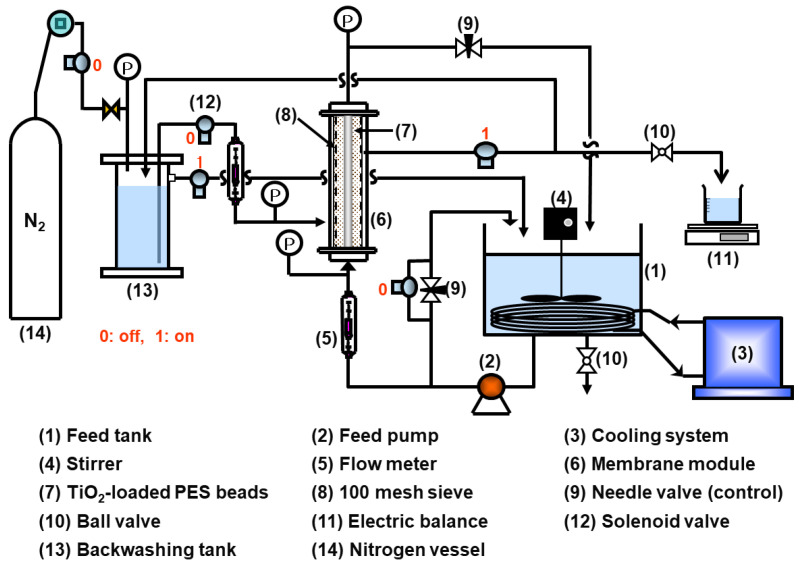
The combined water treatment system of the tubular MF membrane and titanium dioxide-immobilized PES beads with intermittent water backwashing and UV radiation.

The combined water treatment system (6) of the ceramic MF membrane and the TiO_2_-immobilized PES beads (7) instead of PP beads, which was applied in former research [34], is presented in Figure 1. For reducing the membrane fouling, an intermittent water backwashing was achieved by the water treated in the combined water treatment system. The PES beads were fluidizing in the combined module (6). The feed tank (1) kept 10 L of the artificial water of HA and kaolin. To reserve a persistent water viscosity, temperature control water circulator (3) (Model 1146, VWR, Suwanee, GA, USA) maintained the feed water temperature constantly. A stirrer (4) continuously mixed the feed water for uniform concentration in the feed tank, and a pump (2) (Procon, Standex Co., Salem, NH, USA) pushed the feed water into the MF membrane inside. The feed flow rate into the combined module was checked by flowmeter (5) (NP-127, Tokyo Keiso, Tokyo, Japan). The flow rate and trans-membrane pressure (TMP) could be controlled by the valves (9) of the bypass pipe of the pump (2) and the concentrate pipe. The mass of treated water, treated by the combined module, was checked by an electric balance (11) (Ohaus, Canton, MA, USA). The backwashing tank (13) was filled with the treated water when it was not corrected. The treated water was circulated to the feed tank (1) when the water backwashing tank (13) was filled to a maximal level to keep a persistent feed concentration during the experiment. After finishing the three hours’ experiment, physical cleaning by a small brush was performed inside the membrane tube. For estimating the resistances of irreversible and reversible membrane fouling, the treated water flux was checked after the physical cleaning. 

**Figure 2:**
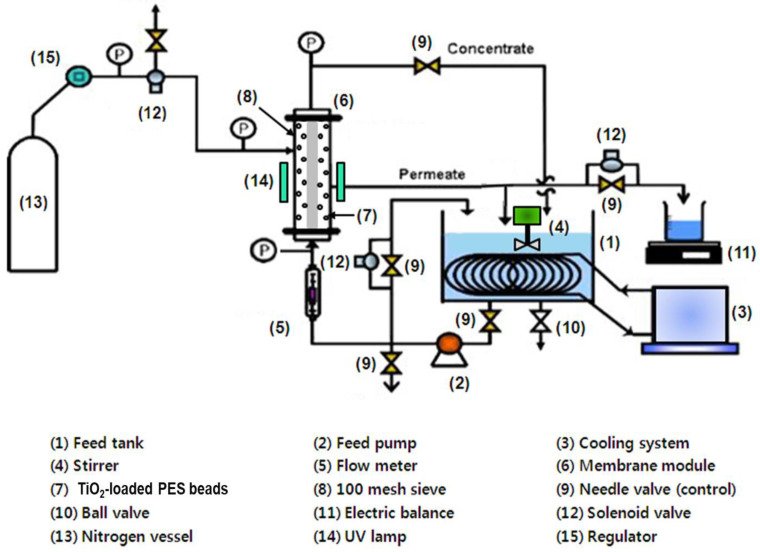
The combined water treatment system of a tubular ceramic MF membrane and titanium dioxide-immobilized PES beads with intermittent air backwashing and UV radiation.

Figure 2 is the combined water treatment system with intermittent air backwashing and UV radiation, which is similar to Figure 1, except for only the nitrogen vessel without a water backwashing tank (13). This combined system with air backwashing was applied in former research [34].

**Scheme 1:**
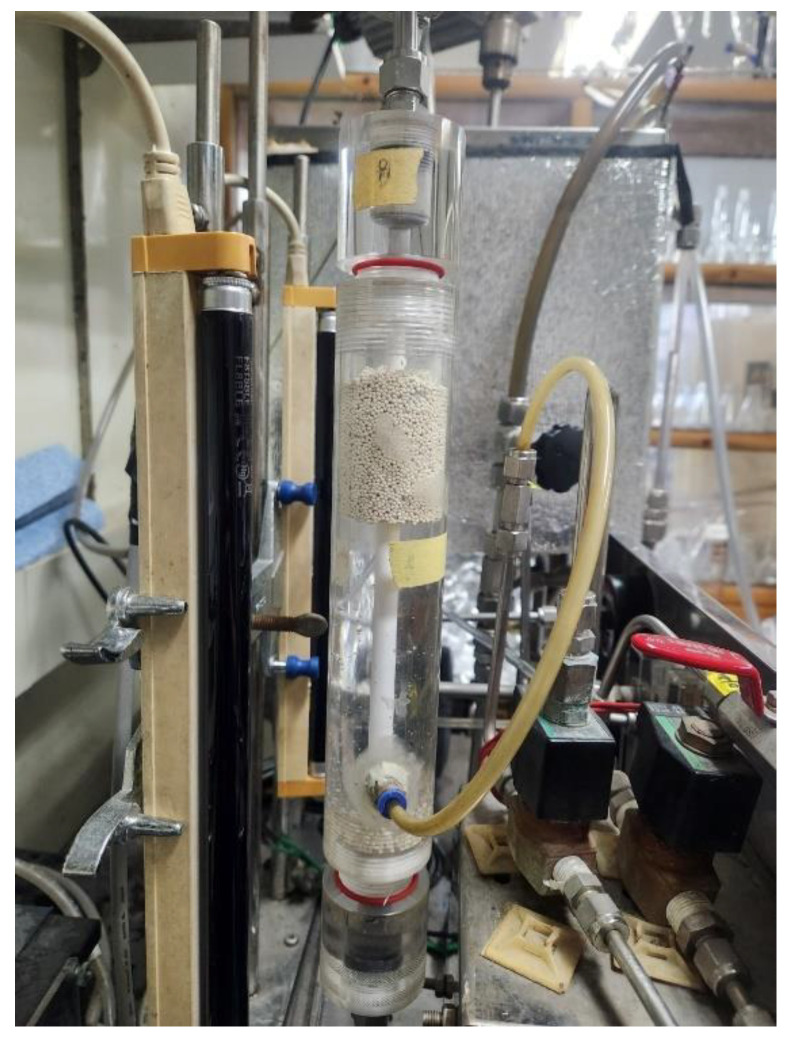
Titanium dioxide-immobilized PES beads in the combined module of an alumina microfiltration membrane and PES beads.

Scheme 1 displays the titanium dioxide-immobilized PES beads in a combined module of alumina microfiltration membrane and PES beads applied in the combined water treatment system. The density of TiO_2_-immobilized PES beads was lower than that of water, and the PES beads were gathered at the top of module.

**Scheme 2:**
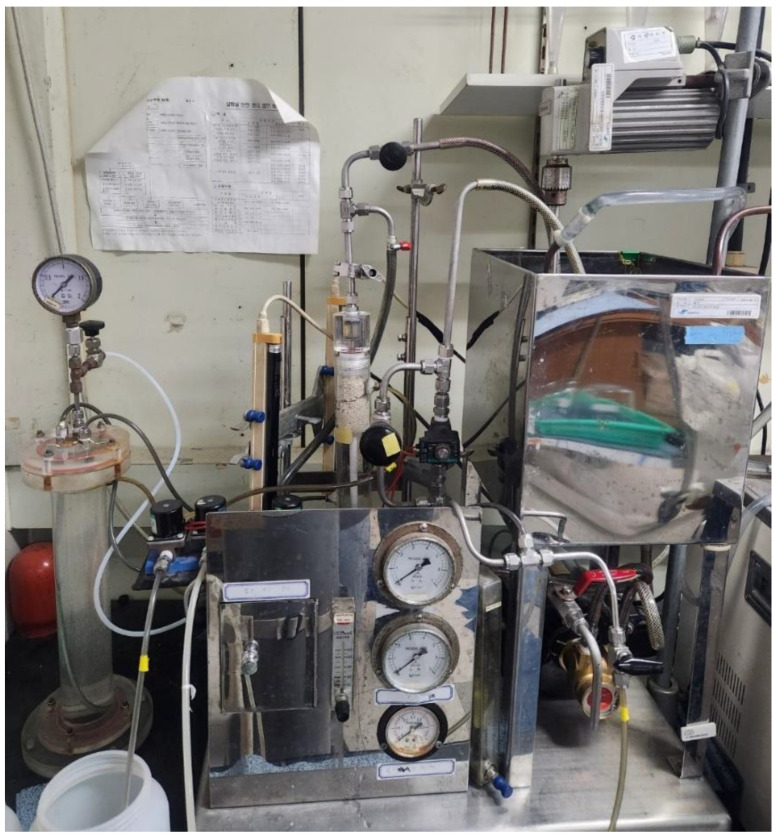
The combined water treatment system of the tubular MF membrane and titanium dioxide-immobilized PES beads with intermittent water backwashing and UV radiation.

Scheme 2 shows the combined water treatment system of tubular MF membrane and titanium dioxide-immobilized PES beads with intermittent water backwashing and UV radiation.

**Scheme 3:**
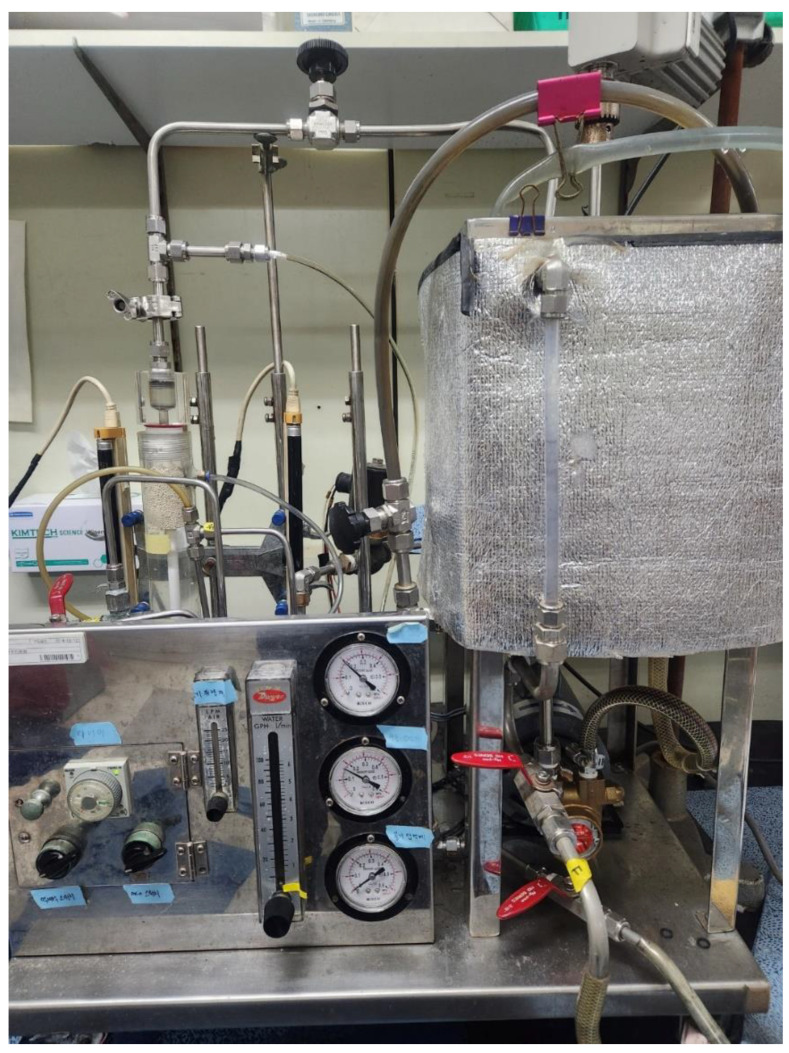
The combined water treatment system of the tubular MF membrane and titanium dioxide-immobilized PES beads with intermittent air backwashing and UV radiation.

Scheme 3 presents the combined water treatment system of a tubular MF membrane and titanium dioxide-immobilized PES beads with intermittent air backwashing and UV radiation.

The filtration time (FT), which was the water backwashing period, was fixed at 10 min, and the backwashing time (BT) at 10 s. Kaolin and HA were fixed at 30 mg/L and 10 mg/L in all of the research, individually. The treated water flux (J) was tested continuously during each research condition during the total 3 h process. In the combined system of tubular ceramic MF, the water or air backwashing pressure was fixed at 2.5 bar, transmembrane pressure (TMP) at 1.8 bar, and the feed flow rate at 1.0 L/min. To maintain a constant viscosity of water in all experiments, the feed water temperature was set at 20 °C. The PES beads’ concentration was changed from 0 to 50 g/L in the combined module space.

The quality of feed and treated water was analyzed every 30 min during each experiment for calculating the treatment efficiencies of turbid matters and DOM. Turbidity was checked by a turbidimeter (2100N, Hach, Loveland, CO, USA), and UV_254_ absorbance was examined by a UV spectrophotometer (Genesys 10 UV, Thermo, Waltham, MA, USA) for determining turbid matters and DOM. The detection limits of the turbidimeter and UV spectrophotometer were 0~4000 NTU (±0.001 NTU) and −0.1~3.0 cm^−1^ (±0.001 cm^−1^), respectively. Before testing UV_254_ absorbance, each sample was filtered by a 0.2 μm syringe filter to reject turbid matters.

After finishing each experiment, all of the synthetic solution was discharged from the combined water treatment system, and distilled water was circulated in the line of the system for 15 min for cleaning. The PES beads were recovered, and the ceramic membrane was separated from the module. The physical cleaning was performed by brushing inside the membrane, and the treated water flux of pure water was checked to decide the irreversible membrane fouling. The combustion at a 550 °C furnace for 30 min could eliminate most of the fouling constituents inside the ceramic membrane. After dropping the membrane temperature, it was dipped in a sodium hydroxide (NaOH) solution of 0.25 N for 3 h, and in a nitric acid (HNO_3_) of 15% for 24 h to melt out inorganic or organic pollutants lingering inside the membrane. For washing and eliminating air inside the membrane pore, it was retained in distilled water for 24 h. Before performing the next study, the water-treated water flux (J_w_) was checked for valuing of the membrane recovery when a cleaning process without any backwashing was accomplished with distilled water. The membrane was recovered enough if the J_w_ was obtained in 95–105% of the new membrane, and it was used for another study. To decrease the effect of the membrane condition on the treatment effectiveness, the recovered membrane was utilized in all of the research. 

## 3. Results and Discussion

The roles of backwashing media (water, air) and TiO_2_ photocatalyst-immobilized PES beads were studied in the combined water treatment system of a tubular (NCMT-7231) ceramic MF membrane and PES beads with UV radiation and intermittent water or air backwashing. Applying the resistance-in-series filtration theory (J = ΔP/(R_m_ + R_b_ + R_f_)) as the equivalent means utilized in the former results [29], where ΔP is TMP, resistances of the membrane, boundary layer, and membrane fouling (R_m_, R_b_, R_f_) were calculated from treated water flux (J) data. For a fresh membrane, the theory was shortened to J = ΔP/R_m_ because the R_m_, R_b_, and R_f_ were zero. Finally, the R_m_ could be decided from the J value for a fresh membrane. The theory was reformed to J = ΔP/(R_m_ + R_b_) at the starting time for the solution prepared with HA and kaolin, and R_b_ could be decided from starting the J (J_0_) and R_m_ data. Furthermore, resistances of the irreversible and reversible membrane fouling (R_if_, R_rf_) could be estimated from the J data, before and after physical cleaning, by applying a brush inside the membrane tube.

### 3.1. Role of TiO_2_-Immobilized PES Beads on Membrane Fouling and Treatment Effectiveness with Intermittent Air Backwashing

**Figure 3:**
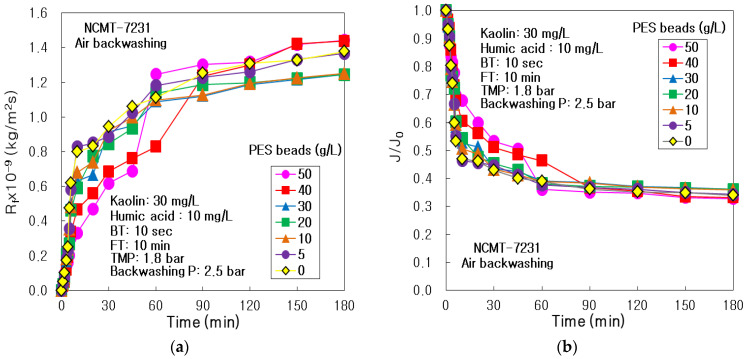
Role of TiO_2_-immobilized PES beads in the combined system of tubular ceramic MF (NCMT-7231) and PES beads with UV radiation and intermittent air backwashing: (**a**) Resistance of membrane fouling; (**b**) Dimensionless treated water flux.

The role of TiO_2_-immobilized PES beads on membrane fouling and treatment effectiveness with air backwashing was investigated by changing the PES beads’ concentration from 0 to 50 g/L for the solution of HA 10 mg/L and kaolin 30 mg/L. The resistances of membrane fouling (R_f_) maintained the bottom data at 50 g/L of the PES beads until 45 min of the process and suddenly increased at 60 min; however, it kept the peak continuously at 0 g/L of PES beads throughout all of the process, and the bottom at 30 g/L of the PES beads after 90 min, as shown in Figure 3a. It verified that the peak PES beads’ concentration of 50 g/L in our experimental condition could be effective in reducing the membrane fouling until 45 min into the process; however, 30 g/L of the PES beads was the optimum after 90 min in this combined water treatment system of tubular ceramic MF and PES beads with intermittent air backwashing. Too many TiO_2_-immobilized PES beads could be coated rapidly by the humic acid and kaolin until 60 min into the process, and then did not have the role of TiO_2_ anymore after 60 min. Finally, the optimal PES bead condition could be 30 g/L for a long time during the process with air backwashing.

In the former work [35] regarding a combined water treatment system of seven channels with ceramic MF (HC04, 0.4 μm) and pure PP beads with air backwashing, the R_f_ obtained the peak at 0 g/L of pure PP beads, and the bottom at 50 g/L for 3 *h*. This means that the more PP beads could repress the membrane fouling, the more remarkable for this combined water treatment system. It verified that the best media concentration was dependent on the materials of the beads in the combined water treatment system with air backwashing.

As shown in Figure 3b, the dimensionless treated water flux (J/J_0_), where J_0_ was the starting treated water flux expected by utilizing the starting two values by an extrapolation means, was paralleled to inspect the role of PES beads for relative decline of treated water flux. The J/J_0_ data overlapped at almost every PES bead concentration; however, those obtained the peak at 50 g/L of the PES bead until the 45 min process; however, it was the peak at 30 g/L of the PES bead after 90 min which detailed experimental data. In the former results [35] regarding the combined system of seven channels with ceramic MF and pure PP beads with air backwashing, the J/J_0_ obtained the maximum at 50 g/L of the PP beads during the 3 h process, and the minimum at 0~10 g/L.

As summarized in Table 3, the final J after the 3 h process (J_180_) was at the peak at 20 and 30 g/L of the PES beads. It verified that the treated water flux could maintain the highest data at the best with 20 and 30 g/L PES beads, for the reason that the membrane fouling was repressed effectively at 20 and 30 g/L in the combined water treatment system with intermittent air backwashing. In conclusion, the J_180_/J_0_ after the 3 h process at 20 and 30 g/L of the PES beads obtained the peak 0.361, which was 1.10 times higher than 0.328 at NBW condition. Nevertheless, the total treated water volume (V_T_) acquired the maximal at 7.06 L at 40 g/L of the PES bead, for the reason that J was conserved higher during the 2 to 60 min process than that of 20 and 30 PES bead concentrations, as displayed in Figure 3b. Lastly, the optimum PES bead concentration could be 40 g/L in the combined water treatment system with air backwashing, owing to the extreme V_T_ in this PES bead concentrations. 

In the former study [35] regarding the combined system of seven channels of ceramic MF and pure PP beads with air backwashing, the V_T_ was the maximum at 2.09 L at 30 and 50 g/L of the PP beads because J was greater preserved during 3 *h* process at 30 g/L of the PP beads. It verified that the best condition of PP beads could be 30 g/L, owing to the maximum J_180_/J_0_ and V_T_. The best condition of PES beads and PP beads did not agree with each other, owing to dissimilar material beads.

As paralleled in Table 3, the resistance of boundary layer (R_b_), that was made by concentration polarization on the surface of the membrane, was the maximum at 20 g/L of the PES beads in the combined system of tubular ceramic MF and TiO_2_-immobilized PES beads. It verified that 20 g/L of the PES bead could diminish the concentration polarization on the surface of the membrane the most powerfully. The R_f,180_ after the 3 h process at 50 g/L of the PES bead obtained the maximum at 1.443 × 10^9^ kg/m^2^s, which was 1.16 times higher than the minimum at 1.245 × 10^9^ kg/m^2^s at 20 and 30 g/L of the PES bead with air backwashing. 

**Figure 4:**
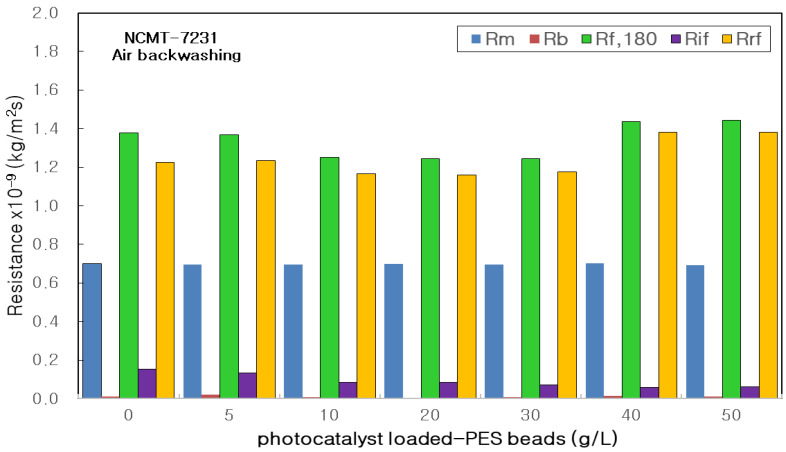
Role of TiO_2_-immobilized PES beads on resistances of membrane, boundary layer, final, irreversible, and reversible membrane fouling (R_m_, R_b_, R_f,180_, R_if_, R_rf_) in the combined system of tubular ceramic MF (NCMT-7231) and PES beads with UV radiation and intermittent air backwashing.

In Figure 4, resistances of membrane, boundary layer, final, irreversible, and reversible membrane fouling (R_m_, R_b_, R_f,180_, R_if_, R_rf_) are visibly paralleled as bar graphs. As the R_rf_ obtained a dominant portion of R_f,180_, it could become a main membrane fouling in this combined water treatment system with air backwashing. This means that a major part of membrane fouling could be easily recovered by physical washing such as a bushing. In addition, the R_rf_ and R_if_ were the minimum at 20 and 40 g/L of the PES beads, respectively. It verified that the 20 and 40 g/L PES beads could diminish the reversible and irreversible membrane fouling effectively in this PES bead scope, respectively.

The treatment efficiencies of turbidity obtained a diminishing tendency when reducing the PES beads from 50 to 0 g/L, as displayed in Table 4. This means that more TiO_2_-coated PES beads could remove turbid matters, such as kaolin, more effectively in the combined water treatment system of tubular ceramic MF and PES beads with air backwashing. In the former results [34] for a combined system of seven channels of ceramic MF and pure PP beads with air backwashing, the turbidity treatment efficiencies obtained a decreasing tendency, with a decrease in PP beads’ concentration; nevertheless, it was the maximum of 95.7% at 50 g/L of the PP beads. These results exactly agreed with the results of this research, in spite of dissimilar material beads.

As paralleled in Table 5, the treatment effectiveness of UV_254_ absorbance, which could replace the DOM concentration, decreased dramatically when declining the PES bead from 50 to 0 g/L. The high input concentration of titanium dioxide-immobilized PES beads in this hybrid module could decompose the humic acid more efficiently by photo-oxidation of UV radiation in the combined water treatment system. It verified that the best PES bead condition could be 50 g/L for DOM treatment in the experimental scope of PES beads. In the former research [34] for the combined system of seven channels of ceramic MF and pure PP beads with air backwashing, the DOM treatment effectiveness did not show a special tendency such as amplifying the PP beads’ concentration because only pure PP beads could not successfully decay the humic acid by adsorption; nevertheless, there was the peak of 56.8% at 40 g/L of the PP beads and a decrease to the minimum of 37.8% at 50 g/L. The DOM treatment efficiencies were much lower than those of this result, owing to just pure PP beads.

### 3.2. Role of TiO_2_-Immobilized PES Beads on Membrane Fouling and Treatment Effectiveness with Water Backwashing

**Figure 5:**
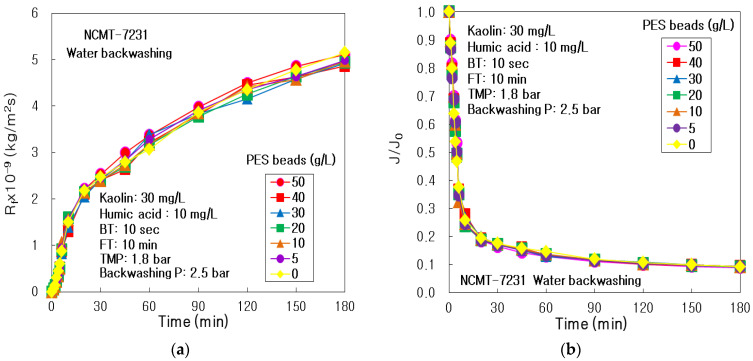
Role of TiO_2_-immobilized PES beads in the combined system of tubular ceramic MF (NCMT-7231) and PES beads with UV radiation and intermittent water backwashing: (**a**) Resistance of membrane fouling; (**b**) Dimensionless treated water flux.

To examine the role of TiO_2_-immobilized PES beads on membrane fouling and treatment effectiveness, the R_f_ was compared depending on the PES bead concentration with water backwashing in the combined system of tubular ceramic MF (NCMT-7231) and TiO_2_-immobilized PES beads with water backwashing, as presented in Figure 5a. The R_f_ data overlapped almost during the whole 3 h process. As compared in Table 6, the R_f,180_ obtained the peak at 0 g/L, and the bottom at 40 g/L PES bead, respectively. Finally, the 40 g/L of the PES bead could be the best condition in the combined system of tubular ceramic MF and TiO_2_-immobilized PES beads with water backwashing. It was a bit of a dissimilar tendency, paralleled with the optimal PES bead with air backwashing which could be 30 g/L for a long process, owing to a dissimilar backwashing medium, as presented in Figure 5a.

In our former research [34] for a combined water treatment system of seven channels of ceramic MF (HC10, 1.0 μm) and pure PP beads with water backwashing, the R_f_ obtained the peak at 50 g/L of the PP beads and the bottom at 5 g/L during the 3 h process. This means that the best PP beads’ concentration could be 5 g/L to control the membrane fouling and high treated water flux in this combined system of 7-channel ceramic MF and PP beads.

As displayed in Figure 5b to examine the role of TiO_2_-immobilized PES beads in relative treated water flux, the J/J_0_ data overlapped almost during all of the 3 *h* process, which was similar with the tendency of R_f_ in Figure 5a. As summarized in Table 6, the final J_180_/J_0_ after the 3 *h* process at 40 g/L of the PES bead obtained the maximum at 0.0927, which was 1.04 times higher than the minimum 0.0888 at 50 g/L of the PES beads. It verified that the best PES bead concentration could be 40 g/L to preserve the peak treated water flux because too many PES beads could not effectively reduce the membrane fouling by blocking UV radiation to the PES bead positioned at the module inside. However, the peak J_180_/J_0_ 0.361 at air backwashing was 3.89 times higher than the maximum at 0.0927 at water backwashing. It verified that air backwashing could be more effective in reducing the membrane fouling than the water backwashing in this combined water treatment. 

In the former study [34] regarding the combined water treatment system of seven channels of ceramic MF and pure PP beads with water backwashing, the J/J_0_ continued greater until 90 min at PP beads 5 g/L than the data at other PP beads’ condition, and obtained the bottom at 50 g/L of the PP beads after 60 min. The values of J_0_ and J_180_ declined at 40 g/L and 50 g/L, respectively, when increasing PP beads’ concentration, because the R_b_ and R_f_ amplified at 40 g/L and 50 g/L of the PP beads, respectively. Lastly, the J_180_/J_0_ after 3 h of the process at 0 g/L of the PP beads was the peak. Nevertheless, the V_T_ was the peak at 5 g/L of PP beads because J continued greater throughout the process than the data of other PP bead conditions.

In conclusion, the peak of V_T_ was 2.67 L at 40 g/L of the PES beads, because the treated water flux could be maintained highly throughout the 3 h process. The maximum V_T_ 7.06 L at air backwashing was 2.64 times higher than the peak of V_T_ 2.67 L at water backwashing. This means that the air backwashing system could acquire more treated water than the water backwashing system. As arranged in Table 6, the R_f,180_ obtained the peak of 5.151 × 10^9^ kg/m^2^s at 0 g/L of the PES beads, which was 1.06 times higher than the bottom at 4.862 × 10^9^ kg/m^2^s at 40 g/L of the PES beads. This verified that the best PES beads could be 40 g/L because the PES beads could reduce the membrane fouling by UV photo-oxidation; nevertheless, too many PES beads blocked UV radiation to the PES beads positioned at the module inside.

**Figure 6:**
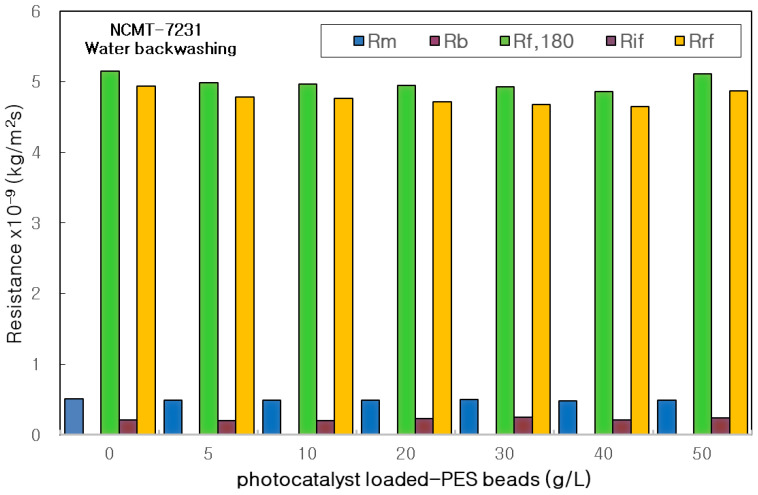
Role of TiO_2_-immobilized PES beads on resistances of membrane, boundary layer, final, irreversible, and reversible membrane fouling (R_m_, R_b_, R_f,180_, R_if_, R_rf_) in the combined system of tubular ceramic MF (NCMT-7231) and PES beads with UV radiation and intermittent water backwashing.

Figure 6 displays all of the resistances to inspect each portion of the total resistance. The R_rf_ presented the main resistance of total membrane fouling; however, the R_if_ was a trivial one, as it had an analogous tendency with the consequences of air backwashing as shown in Figure 4 of Section 3.1. The difference of R_f,180_, R_if_, and R_rf_ was little, depending on the PES bead concentration; however, the data regarding R_f,180_, R_if_, and R_rf_ obtained much higher results than those of air backwashing. The R_if_ was at the bottom at 5 g/L of the PES beads for water backwashing, which was 3.43 times higher than the minimum at 40 g/L of the PES beads for air backwashing. This verified that the air backwashing could be more efficient than water backwashing in this combined water treatment system.

As paralleled in Table 7, the treatment effectiveness of turbidity obtained almost constant from 97.0% to 98.1%; however, the peak was 98.1% at 40 g/L and the bottom was 97.9% at 0 g/L of the PES beads. Lastly, the best PES bead could be 40 g/L to remove the turbid matter in this combined water treatment with water backwashing. In the former results [34] regarding the combined water treatment system of seven channels of ceramic MF and pure PP beads using water backwashing, the treatment effectiveness of turbidity continued nearly persistent in the scope of 97.5% and 98.9%, despite the pure PP beads’ condition. This verified that the turbid substances could be removed successfully by the ceramic membrane only, independent of the PP beads’ condition in this combined system.

As displayed in Table 8, the treatment effectiveness of DOM reduced significantly when decreasing the PES beads, except those 50 g/L with water backwashing. The maximum effectiveness obtained was 82.3% at 40 g/L, and the minimum was 77.8% at 0 g/L PES beads. This means that the best PES bead could be 40 g/L to remove DOM in the combined system with water backwashing. 

In the former work [34] regarding the combined water treatment system of seven channels of ceramic MF and pure PP beads using water backwashing, the treatment effectiveness of DOM did not present a uniform tendency; nevertheless, it obtained the peak of 51.3% at 5 g/L of PP beads. This verified that the best PP beads’ concentration could be 5 g/L to reduce DOM in this combined system of MF and the pure PP beads.

**Figure 7:**
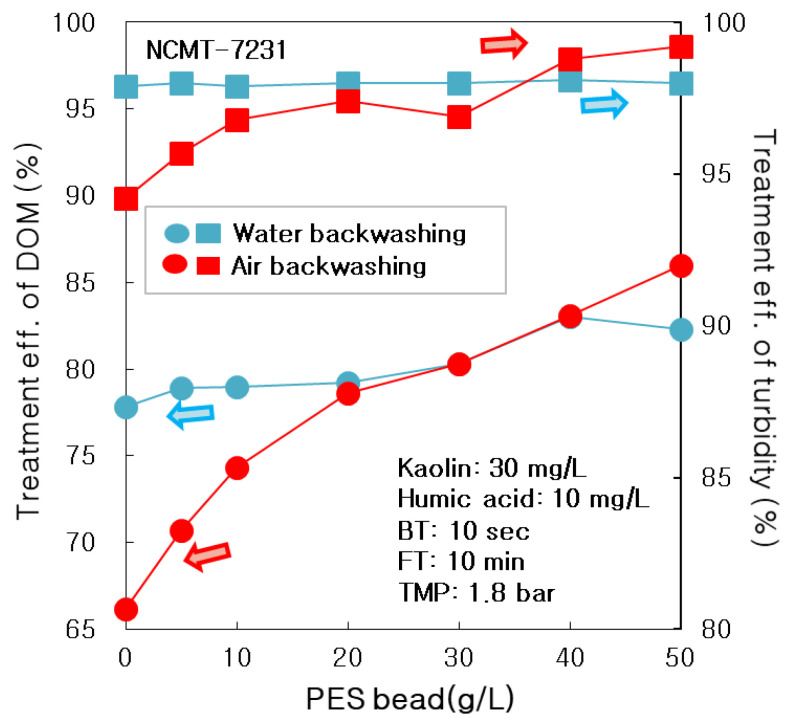
Role of TiO_2_-immobilized PES beads on treatment effectiveness of turbidity and DOM in the combined system of a tubular ceramic MF (NCMT-7231) and PES beads using UV radiation and intermittent water and air backwashing.

In Figure 7, the treatment efficiencies of turbidity and DOM were compared, depending on PES beads for water and air backwashing in the combined water treatment system. The effectiveness of turbidity for air backwashing obtained an increasing tendency when increasing PES beads; however, for water backwashing, it obtained almost constant, independent of PES beads. The effectiveness of DOM for air backwashing increased dramatically when increasing PES beads; nevertheless, for water backwashing, it increased slowly and obtained a maximum at 40 g/L PES beads.

**Scheme 4:**
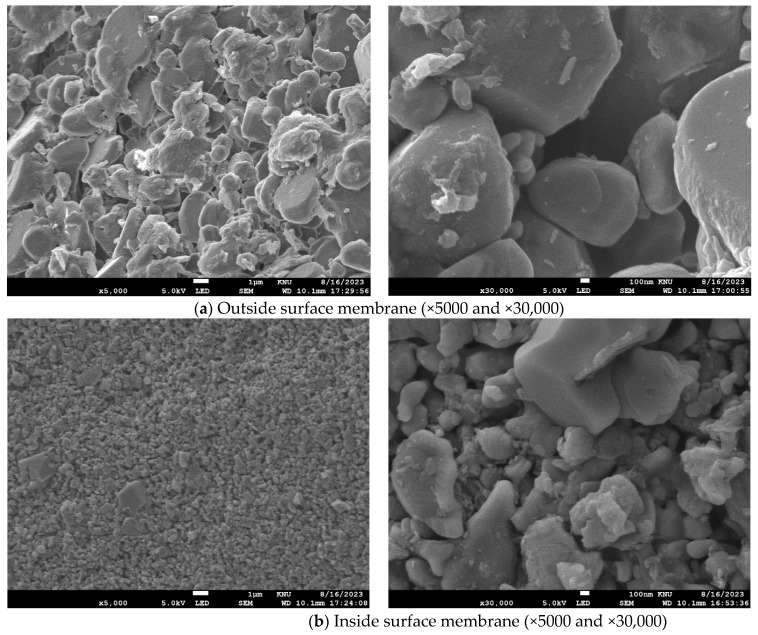
SEM (Scanning Electron Microscope) pictures of the alumina membrane surface: (**a**) Outside surface membrane, (**b**) Inside surface membrane.

Scheme 4 shows the SEM (Scanning Electron Microscope) pictures of the alumina membrane surface after operating several times in this combined water treatment system. The outer surface of the used membrane was almost clean, and the membrane pore could be distinguished. However, the inner surface was accumulated by kaoline particles and the membrane pore was blocked by contaminated particles because the combined module was operated as an in-and-out type, which means the feed flowed inside the membrane tube.

## 4. Conclusions

In this research, the role of TiO_2_-immobilized PES beads for air and water backwashing was studied in the combined water treatment system of tubular ceramic MF and PES beads. The results of TiO_2_-immobilized PES beads were associated with those of the former study [32,33] in the combined system of the 7-channel ceramic MF and pure PP beads. In conclusion, the following results could be drawn from this research.

(1) For air backwashing, the resistances of membrane fouling (R_f_) sustained the bottom data at 50 g/L PES beads up until 45 min of the process, and suddenly increased at 60 min; however, it was the bottom at 30 g/L of the PES beads after 90 min. Too many TiO_2_-immobilized PES beads could be coated rapidly by the humic acid and kaolin up until 60 min of the process, and then did not have the role of TiO_2_ anymore after 60 min. Finally, the optimal PES bead condition could be 30 g/L for long time during the process. In our former work [35] regarding the combined water treatment system of seven channels of ceramic MF and pure PP beads with air backwashing, the R_f_ obtained the bottom at 50 g/L PP beads. This means that more PP beads could repress the membrane fouling the more remarkably in this combined water treatment system. This also verified that the best media condition was dependent on the material of the beads in the combined water treatment system with air backwashing.
(2) The dimensionless treated water flux (J_180_/J_0_) after the 3 h process at 20 and 30 g/L of the PES beads obtained the peak; nevertheless, the total treated water volume (V_T_) had the peak at 40 g/L of the PES beads. Finally, the best PES bead concentration could be 40 g/L in the combined water treatment system with air backwashing. In the former research [35], the V_T_ was the maximum at 30 and 50 g/L of the PP beads. This means that the best condition of PP beads could be 30 g/L. The best condition of PES and PP beads did not agree with each other, owing to different material beads.
(3) For water backwashing, the R_f,180_ obtained the bottom at 40 g/L PES beads. Finally, the 40 g/L PES beads could be the best condition. It was a little dissimilar tendency, when compared to the optimal PES beads with air backwashing could be 30 g/L for long process, owing to a different backwashing medium. In our former results [34] regarding the combined water treatment system of seven channels of ceramic MF and pure PP beads, the R_f_ was at the bottom at 5 g/L of the PP beads during the 3 h process. This verified that the best PP beads’ concentration could be 5 g/L to reduce the membrane fouling and a lot of treated water volume.
(4) The peak V_T_ was 2.67 L at 40 g/L PES beads because the treated water flux could be maintained extremely throughout the 3 h process. The maximum V_T_ 7.06 L at air backwashing was 2.64 times higher than the peak V_T_ 2.67 L at water backwashing. This means that the air backwashing system could acquire more treated water than the water backwashing system.
(5) The effectiveness of turbidity for air backwashing obtained an increasing tendency when increasing PES beads; however, for water backwashing, it obtained almost constant, independent of PES beads. The effectiveness of DOM for air backwashing increased dramatically when increasing the beads; however, for water backwashing, it increased slowly and obtained a maximum at 40 g/L PES beads.

## Figures and Tables

**Table 1:** Characteristics of the tubular ceramic MF membrane utilized in this study.

Membrane Model	NCMT-7231
Pore size (μm)	0.4
No. of channels	1
Outer diameter (mm)	8
Inner diameter (mm)	6
Length (mm)	254
Surface area (cm^2^)	47.9
Material	⍺-alumina
Company	Nanopore Inc. (Cheonan, Republic of Korea)

**Table 2:** Characteristics of the TiO_2_-immobilized beads applied in this study.

Material of the beads	Polyethersulfone (PES)
TiO_2_ coating method	Phase inversion
Diameter (mm)	1.4–1.8
Weight (mg)	1.3–1.8
Average weight (mg)	1.5

**Table 3:** Role of TiO_2_-immobilized PES beads on filtration elements in the combined system of tubular ceramic MF (NCMT-7231) membrane and PES beads with intermittent air backwashing.

PES Bead (g/L)	50	40	30	20	10	5	0
R_m_ × 10^−9^ (kg/m^2^s)	0.694	0.701	0.695	0.700	0.695	0.694	0.699
R_b_ × 10^−9^ (kg/m^2^s)	0.011	0.016	0.008	0.003	0.007	0.021	0.012
R_f,180_ × 10^−9^ (kg/m^2^s)	1.443	1.438	**1.245**	**1.245**	1.251	1.367	1.378
R_if_ × 10^−9^ (kg/m^2^s)	0.062	**0.058**	0.070	0.085	0.083	0.133	0.153
R_rf_ × 10^−9^ (kg/m^2^s)	1.381	1.380	1.175	**1.161**	1.167	1.233	1.225
J_0_ (L/m^2^h)	902	887	903	904	905	888	893
J_180_ (L/m^2^h)	296	295	**326**	**326**	325	305	304
J_180_/J_0_	0.328	0.332	**0.361**	**0.361**	0.359	0.344	0.340
V_T_ (L)	7.03	**7.06**	6.96	6.91	6.89	6.56	6.53

Bold numbers represent the best condition in our experimental range.

**Table 4:** Water quality and treatment effectiveness of turbidity in the combined system of tubular ceramic MF (NCMT-7231) and PES beads with UV radiation and intermittent air backwashing for the role of TiO_2_-immobilized PES beads.

PES Bead (g/L)	Turbidity (NTU)				Average Treatment Effectiveness (%)
	Feed Water		Treated Water		
	Scope	Average	Scope	Average	
50	35.2~37.6	36.8	0.283~0.374	0.312	99.2
40	35.8~37.4	36.8	0.358~0.638	0.480	98.7
30	34.4~36.8	35.6	1.009~1.332	1.119	96.9
20	35.2~37.4	36.5	0.776~1.033	0.909	97.5
10	36.1~37.1	36.6	0.978~1.351	1.179	96.8
5	35.4~37.3	36.6	1.429~1.715	1.586	95.7
0	35.8~37.1	36.5	1.853~2.384	2.099	94.2

**Table 5:** Water quality and treatment effectiveness of DOM (UV_254_ absorbance) in the combined system of tubular ceramic MF (NCMT-7231) and PES beads with UV radiation and intermittent air backwashing for the role of TiO_2_-immobilized PES beads.

PES Bead (g/L)	UV_254_ Absorbance (cm^−1^)				Average Treatment Effectiveness (%)
	Feed Water		Treated Water		
	Scope	Average	Scope	Average	
50	0.251~0.274	0.265	0.021~0.061	0.037	86.0
40	0.253~0.271	0.264	0.021~0.051	0.038	85.6
30	0.258~0.281	0.267	0.036~0.071	0.053	80.3
20	0.253~0.276	0.263	0.029~0.081	0.058	78.0
10	0.259~0.277	0.268	0.032~0.103	0.069	74.3
5	0.258~0.278	0.266	0.036~0.103	0.078	70.7
0	0.253~0.271	0.263	0.076~0.105	0.088	66.2

**Table 6:** Role of TiO_2_-immobilized PES beads on filtration elements in the combined system of a tubular ceramic MF (NCMT-7231) membrane and PES beads with intermittent water backwashing.

PES Bead (g/L)	50	40	30	20	10	5	0
R_m_ × 10^−9^ (kg/m^2^s)	0.493	0.486	0.497	0.488	0.492	0.491	0.509
R_b_ × 10^−9^ (kg/m^2^s)	0.005	0.010	0.005	0.007	0.011	0.011	**0.015**
R_f,180_ × 10^−9^ (kg/m^2^s)	5.113	**4.862**	4.926	4.945	4.967	4.985	5.151
R_if_ × 10^−9^ (kg/m^2^s)	0.244	0.213	0.248	0.228	0.200	**0.199**	0.209
R_rf_ × 10^−9^ (kg/m^2^s)	4.869	**4.649**	4.678	4.717	4.767	4.786	4.942
J_0_ (L/m^2^h)	1275	1278	1264	1282	1263	1263	1213
J_180_ (L/m^2^h)	113	**119**	117	117	116	116	112
J_180_/J_0_	0.0888	**0.0927**	0.0926	0.0910	0.0919	0.0916	0.0922
V_T_ (L)	2.57	**2.67**	2.65	2.64	2.63	2.60	2.58

Bold numbers represent the best conditions in our experimental range.

**Table 7:** Water quality and treatment effectiveness of turbidity in the combined system of a tubular ceramic MF (NCMT-7231) membrane and PES beads with intermittent water backwashing for the role of TiO_2_-immobilized PES beads.

PES Bead (g/L)	Turbidity (NTU)				Average Treatment Effectiveness (%)
	Feed Water		Treated Water		
	Scope	Average	Scope	Average	
50	33.5~34.3	33.9	0.620~0.708	0.664	98.0
40	33.4~34.7	34.1	0.592~0.688	0.638	98.1
30	35.2~36.2	35.7	0.652~0.758	0.706	98.0
20	35.2~36.1	35.7	0.692~0.758	0.722	98.0
10	34.0~35.1	34.6	0.687~0.762	0.722	97.9
5	33.8~35.9	34.8	0.654~0.786	0.707	98.0
0	35.4~37.2	36.3	0.689~0.839	0.758	97.9

**Table 8:** Water quality and treatment effectiveness of DOM (UV_254_ absorbance) in the combined system of a tubular ceramic MF (NCMT-7231) membrane and PES beads using intermittent water backwashing for the role of TiO_2_-immobilized PES beads.

PES Bead (g/L)	UV_254_ Absorbance (cm^−1^)				Average Treatment Effectiveness (%)
	Feed Water		Treated Water		
	Scope	Average	Scope	Average	
50	0.257~0.265	0.261	0.042~0.050	0.046	82.3
40	0.250~0.261	0.256	0.038~0.048	0.044	83.0
30	0.262~0.271	0.266	0.048~0.057	0.053	80.3
20	0.256~0.265	0.260	0.050~0.059	0.054	79.2
10	0.253~0.266	0.260	0.050~0.060	0.055	79.0
5	0.251~0.264	0.258	0.047~0.060	0.055	78.9
0	0.255~0.266	0.260	0.052~0.061	0.058	77.8

## Data Availability

Data is unavailable due to privacy or ethical restrictions.
